# Quarterly Report of the Weather and Diseases of Cincinnati

**Published:** 1828-04

**Authors:** 


III. ORIGINAL.

7. *Quarterly report of the Weather and Diseases of Cincinnati.* In commencing a regular series of quarterly reports on our Weather and Diseases, it is proper to say something of the physical and statistical condition of the city.

*Topography.* Cincinnati is built on the north bank of the Ohio river, in N. lat. 39° 6' 30'', and long. W. from Greenwich 84° 24'—from Washington 7° 24'. It covers part of a basin, or expansion of the valley, the area of which—equal to four or five square miles—is divided into two nearly equal parts, by the Ohio, running from east to west. The plain on which the city stands, consists of two terraces—the lower, but a few feet above high water mark; the other about fifty feet more elevated. The elevation of the former above the level of the sea is 495 feet. The hills which surround the City rise about 360 higher. In no direction, for a great distance, is there a single mountain; and the nearest lakes are 200 miles to the north.

*Climate.* From observations continued through eight successive years, the mean annual heat of the city is 54° 25*; but our purpose, at present, is to speak of its winter climate only. The mean temperature of our winters is 32° 94—that of December being 34° 54, of January 29° 88, and of February 34° 42. In seven winters out of eight, the mercury falls, once or oftener, below 0; and, now and then, to–10°–14° and even–18°. On the other hand, it frequently rises, during the same season, above 70°. Continued frost for several weeks is uncommon, though not unprecedented. The prevailing winds are west and north-west. The snows are seldom more than six inches in depth; and, in general, do not lie beyond a few days. Copious rains are by no means uncommon. Some winters are so warm and moist, that the buds of certain trees and shrubs swell and partially unfold themselves even in January; but in other years, vegetation does not manifest itself till the month of March. On the whole, the characteristic feature of the winter climate of Cincinnati is variability; which might be inferred from its latitude, for, across the whole United States, the zone which lies between the 37° and 41° of latitude, has a winter climate compounded of the tem-

*Picture of Cincinnati, p. 93.

perate and the rigorous. *Communis annibus*, we have these varieties in constant alternation; but, occasionally, the whole season is characterised by the mildness of 36° or the severity of 42°.

*Civil Condition.* The dwelling houses of Cincinnati are either of brick or wood. Its inhabitants, even of the poorest classes, are generally well protected from the inclemency of the weather. The fuel, in common use, is wood. The population amounts to about 20,000; more than three-fourths of whom are emigrants from various countries. They are, in general, warmly clad and well fed. Animal food is abundant, and the majority are accustomed to eat it, at least twice a day. Tea and coffee are in universal use; whiskey, strong beer and cider, are common beverages.

*Winter of* 1828. The winter of the present year was remarkable for its great mildness and copious rains. The mercury never fell to zero; and the ground, at no time but slightly frozen, was never covered with snow more than an inch in depth. Indeed, but two or three snow storms occurred, and they were inconsiderable both in violence and duration. The Ohio was, generally, swollen; and not a single sheet of ice at any time formed along its margins at this place, or was seen floating from above. Even in January, a number of plants, which commonly exhibit no vegetation, till about the first of March, began to unfold their buds; and the process continued, steadily but slowly, to advance through the succeeding month; for the adage, that a mild January gives a rigorous February, was not realised. Many persons, indeed, pronounced this winter unprecedented, though, as it respects temperature at least, this was a mistake; for several of the winters immediately preceding that of 1791, were equally mild; but whether as rainy, is not known. Those, also, of 1793, 1796, 1800, 1806, and 1810, not to mention others, were exceedingly temperate. On the whole, however, that of the present year will be deservedly remembered for its warmth and continued humidity.

*Diseases.* A great number of diseases are excited by causes, not connected with local situation, climate, contagions, or insensible morbid states of the atmosphere. Such affections seldom exhibit any tendency to an epidemic character; and, in general, may be passed unnoticed in a report of the kind which we propose to make. Observing this rule, we shall briefly sketch the maladies of the three winter months just passed.

The autumn of 1827 was unusually healthy. Cincinnati, perhaps, never presented fewer cases of autumnal fever, in proportion to its population; nor is there, probably, a town in any of the middle or southern parts of the United States, that was more healthy. The propriety of mentioning this fact, will be perceived by all who, like ourselves, have observed that the diseases of winter are modified by the Epidemic constitution of the preceding autumn. Thus, when that constitution has been typhus, or when it has been highly bilious, the winter diseases which follow, are either typhoid, or complicated with biliary derangements, beyond what they exhibit when autumn has passed by, without leaving on us any special impress. It was in this state that winter found us. At the same time there was no hooping cough, measles, mumps, scarlatina, small-pox, varioloid, or typhus constitution prevalent. Every circumstance was favorable, then, to an unrestricted and unmodified effect of the winter itself. Of its predominant characteristics we have spoken. They were great mildness of temperature and excessive humidity; and no better opportunity is likely to present itself, for observing the morbid effects of this kind of weather upon the inhabitants of a latitude, where, in the winter, it is out of season. We proceed to say, then, that the prevailing diseases of the past winter were inflammations, and these chiefly of the lungs. All the varieties of *Brochitis* frequently presented themselves. This, indeed, was the reigning disease of the winter; in most cases of a purely inflammatory character, and, in general, not complicated with much hepatic derangement. It affected persons of every age. Its prominent symptoms were cough, with an expectoration of mucous, generally tinged with blood; dyspnœa; sense of constriction across the breast; more or less of pleuritic pain, sometimes acute and fixed, at others wandering; lastly, fever, with a pulse in general not well developed. It was commonly frequent and irregular in its rhythm; often small and contracted; now and then palpably intermittent, but, in some instances, full, tense and regular in the succession of strokes. By *auscultation* the respiration was sonorous, and the mucous rattle, for the most part, distinctly audible. In children this afforded a good diagnostic difference between bronchitis and croup; which, taken in connection with the kind of cough, and with the early expectoration, served sufficiently well to distinguish it from that malady. In aged persons it sometimes assumed an acute form; but oftener pursued a chronic course, without much fever, and attended with asthmatic respiration. In some of these cases, the difficulties in the pulmonary circulation were so great, as to produce a plethoric state of the descending cava, with vertigo and other cerebral disturbances. A similar condition of the ascending cava led to irregularities in the portal circulation, and the consequent production of bilious symptoms. In patients of every age, affected either with the acute or chronic form of the disease, when it was far advanced and about to end fatally, the respiration became exceeding quick and frequent; coma supervened, apparently, from cerebral pressure, and the complexion, especially of the nails and epithelium of the lips, assumed a purple teint, from the imperfect æreation of the blood.

As our limits are filled, and this malady still continues to occur, we shall defer an account of its treatment till we report the diseases of spring.

